# Comparison of Periprocedural and Intermediate-Term Outcomes of TAVI in Patients with Ejection Fraction ≤ 20% vs. Patients with 20% < EF ≤ 40%

**DOI:** 10.3390/jcm12062390

**Published:** 2023-03-20

**Authors:** Arber Kodra, Michael Cinelli, Renita Alexander, Rahming Hamfreth, Denny Wang, Shankar Thampi, Craig Basman, Chad Kliger, Jacob Scheinerman, Luigi Pirelli

**Affiliations:** 1 Department of Cardiothoracic Surgery, Lenox Hill Hospital, New York, NY 10075, USA; 2 Department of Cardiology, Staten Island University Hospital, Staten Island, NY 10305, USA

**Keywords:** transcatheter aortic valve replacement, low ejection fraction, inotropes, assist devices

## Abstract

Treatment of congestive heart failure (CHF) with left ventricular (LV) systolic dysfunction and severe aortic stenosis (AS) is challenging, yet transcatheter aortic valve replacement (TAVR) has emerged as a suitable treatment option in such patients. We compared the periprocedural outcomes of TAVR in patients with an ejection fraction (EF) of ≤20% (VLEF group) to patients with an EF > 20% to ≤40% (LEF group). We included patients with severe AS and reduced LV ejection fraction (LVEF ≤ 40%) who underwent TAVR at four centers within Northwell Health between January 2016 and December 2020. Over 2000 consecutive patients were analyzed, of which 355 patients met the inclusion criteria. The primary composite endpoint was in-hospital mortality, moderate or greater paravalvular (PVL), stroke, conversion to open surgery, aortic valve re-intervention, and/or need for PPM. Secondary endpoints were length of stay, NYHA classification at 1 month and 1 year, mortality at 1 month and 1 year, mean valve gradient at 1 month, KCCQ score at 1 month, and ≥ moderate PVL at 1 month. There was no difference in the primary composite endpoint between the two groups (23.6% for VLEF vs. 25.3% for LEF, *p* = 0.29). During TAVR placement, 40% of patients in the VLEF group required ≥1 vasopressors for hypotension lasting ≥30 min vs. only 21% of patients in the LEF group (*p* < 0.01). Intra-aortic balloon pump (IABP) use during procedure was greater in the VLEF group (9% vs. 1%, *p* < 0.01)—all placed post TAVR. Emergency ECMO use was higher in the VLEF group as well (5% vs. 0%). Total length of stay was significantly different between the two groups as well (6 days vs. 3 days, *p* < 0.01). Both groups had a change in LVEF of ~10%. One-year outcomes were similar between the groups. All-cause mortality at 1 year was not significantly different at 1 year (13% for VLEF vs. 11% for LEF), and KCC scores were also similar (77.54 vs. 74.97). Mean aortic valve gradients were also similar (12 mmHg vs. 11 mmHg, *p* = 0.48). Our study suggests that patients with EF ≤ 20% can safely have TAVR with similar periprocedural outcomes compared to patients with EF > 20% to ≤40% despite higher rates of vasopressor and mechanical support.

## 1. Introduction

The management of combined left ventricular (LV) systolic dysfunction and severe aortic stenosis (AS) is challenging. Concomitant AS in addition to congestive heart failure (CHF) results in increased LV load due to a fixed valvular obstruction, which leads to worsening LV systolic pressure, increased ventricular wall stress, and decreased coronary perfusion [1,2,3]. Transcatheter aortic valve replacement (TAVR) has emerged as a suitable alternative to surgical aortic valve replacement (SAVR) in patients with underlying comorbidities such as LV systolic dysfunction [4,5]. Two transcatheter heart valves (THV) were commercially available in the United States at the time of this study: balloon-expandable valves (BEV) (Sapien, Edwards Lifesciences, Irvine, CA, USA) or self-expandable valves (SEV) (Corevalve, Medtronic Inc., Minneapolis, MN, USA). More recently, the self-expandable Portico transcatheter heart valve (Abbott Cardiovascular, Plymouth, MD, USA) was approved by the Food Drug Association (FDA) for high-risk patients. Although TAVR has been shown to be feasible in patients with systolic dysfunction [2], its safety in patients with an ejection fraction ≤20% remains unclear. Furthermore, there are few head-to-head comparisons on type of THV (balloon vs. self-expandable) in patients with very low EF [6]. We therefore evaluated outcomes in patients with severely reduced EF and compared THV selection amongst them.

## 2. Methods

**Figure 1:**
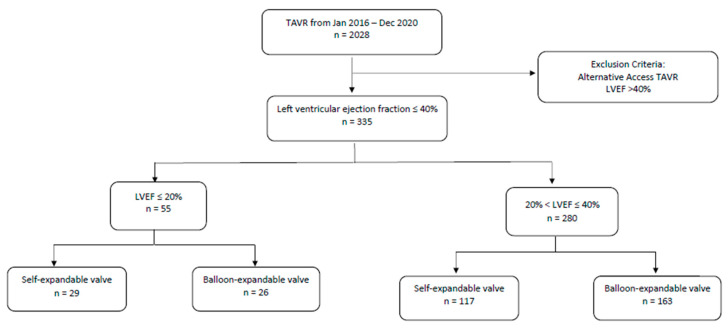
Schematic of study design. LVEF, left ventricular ejection fraction; TAVR, transcatheter aortic valve replacement.

In this retrospective study, we included patients with severe AS and LV systolic dysfunction (LVEF ≤ 40%) who underwent TAVR at four high-volume centers within Northwell Health from January 2016 through December 2020. Data were collected from the Northwell Transcatheter Heart Therapy registry. Study data were collected and managed using REDCap electronic data capture tools hosted at Northwell Health. A total of 2028 consecutive patients were analyzed. Patients with an ejection fraction >40% were excluded, as were patients that required alternative access TAVR. In our database of patients with EF < 40%, only one patient had an alternative access TAVR, which was via a transapical approach. We felt that since this was a hybrid surgical and transcatheter intervention, this patient’s data should be excluded. Ultimately, 335 patients with systolic dysfunction that underwent transfemoral TAVR were included in our study. Fifty-five of these patients had an EF ≤ 20% (referred to as very low EF (VLEF). Patients with systolic dysfunction and an LVEF > 20% were referred to as LEF (Figure 1).

### 2.1. TAVR Procedure

All TAVR cases were planned after contrast-enhanced multidetector computed tomography (MDCT). All MDCT examinations were reviewed by an experienced interventional cardiologist using 3mensio Structure Heart Software, Version 10.0 (3mensio Medical Imaging B.V., Bilthoven, The Netherlands). Cases were performed predominantly with conscious sedation. Only 5% of cases were performed with a transesophageal echocardiogram. A temporary transvenous pacemaker was placed in all cases for rapid pacing during valve deployment and as backup pacing. Rapid pacing was performed for all BEV at 180 bpm and for 55% of SEV at 130–150 bpm. Predilation was performed for all SEV but for only 11% of BEV. A post-valve deployment echocardiogram was performed for all cases.

### 2.2. Baseline Characteristics

Demographics were obtained, including age/gender/STS score. Low-flow, low-gradient (LFLG), severe AS was defined as a discordance between the aortic valve area (AVA) and the mean gradient (MG) (AVA < 1 cm^2^ but MG < 40 mmHg). Dobutamine stress echocardiography or invasive cardiac catheterization ± use of dobutamine infusion was used to confirm LFLG severe AS when necessary. New York Heart Association (NYHA) class was recorded for all patients.

### 2.3. Endpoints

In-hospital adverse events, echocardiographic data, and clinical follow-up at 1 month and 1 year were obtained. The primary composite endpoint was defined as a composite of in-hospital mortality, moderate or greater paravalvular (PVL), stroke, conversion to open surgery, aortic valve re-intervention, and/or need for permanent pacemaker (PPM). Secondary endpoints were length of stay as well as NYHA classification at 1 month and 1 year, mortality at 1 month and 1 year, mean valve gradient at 1 month, Kansas City Cardiomyopathy questionnaire (KCCQ) score at 1 month, and ≥ moderate PVL at 1 month and 1 year.

The periprocedural outcomes that were observed included need for IABP support, need for extracorporeal membrane oxygenation (ECMO), need for transfusion, observed intraoperative hypotension, need for ≥1 pressor for ≥30 min intraoperatively, and need for pre/post-balloon aortic valvuloplasty (BAV). Endpoints were defined as per the Valve Academic Research Consortium (VARC III) definitions.

### 2.4. Statistical Analysis

Statistical analysis for the categorical value of age was performed with a Fisher’s exact test. All continuous variables including number of females, IABP support, ECMO, transfusion, intraoperative hypotension, pressor support intraoperatively, pre/post BAV, need for permanent pacemaker (PPM), stroke, paravalvular leak, discharge survival, and one-month survival were all analyzed using *t*-test analysis, where *p* ≤ 0.05 was considered statistically significant.

## 3. Results

### 3.1. Baseline Characteristics

There was no statistically significant difference in age or STS score among the VLEF and the LEF groups. LFLG severe AS was present in approximately 60% of patients, with no difference among groups. The presence of a cardiovascular implantable electrical device (CIED) or PPM was similar between both groups (40% vs. 29%, *p* = 0.11)). Rates of prior percutaneous coronary intervention (PCI) or coronary artery bypass grafting (CABG) differed in that more patients in the LEF group had prior coronary revascularization (42% vs. 57%, *p* = 0.04). There was also no statistically significant difference in the presence of risk factors such as DM, PVD and smoking, anemia, and renal insufficiency. Preoperative blood work (including hemoglobin and creatinine levels) was similar among groups. KCCQ scores were similar among groups. Baseline AVA, mean gradient, and peak gradient were also similar. (Table 1). The percentage of patients who received a self-expanding valve was numerically higher in the VLEF group but not significantly (53% vs. 42%, *p* = 0.14).

### 3.2. Peri-Procedural Outcomes

There was no difference in the primary composite endpoint between the two groups (23.6% for VLEF vs. 25.3% for LEF, *p* = 0.29). There was no significant difference in procedure-related stroke as well (2% vs. 1%, *p* = 0.59). Rates of conversion to surgery and valve re-intervention were extremely low and not statistically significant. Discharge alive occurred in 98% of patients in both groups (*p* = 0.99). There was no difference in periprocedural PVL greater than mild, although it was numerically higher in the LEF group (2% vs. 5%, *p* = 0.48). PPM post procedure occurred in 18% of patients in the VLEF group and in 17% of patients in the LEF group (*p* = 0.85). There was a statistical difference in postoperative bleeding, with a larger number of patients in the VLEF group requiring transfusions (38% vs. 21%, *p* < 0.01). During TAVR placement, 40% of patients in the VLEF group required ≥1 vasopressors for hypotension lasting ≥30 min vs. only 21% of patients in the LEF group (*p* < 0.01). IABP use during procedure was greater in the VLEF group (9% vs. 1%, *p* < 0.01)—all placed post TAVR. Emergency ECMO was required for one patient in the VLEF group as well (5% vs. 0%). Total length of stay was significantly different between the two groups as well (6 ± 3 days for VLEF vs. 3 ± 2 days, *p* < 0.01) (Table 2).

### 3.3. Clinical Outcomes at One Month and One Year

At 1 month (9% vs. 5%, *p* = 0.19) and 1 year (13% vs. 11%, *p* = 0.64), there was no significant difference in mortality. (Table 3) Mean gradients at 1 month and 1 year were also not statistically significantly different between the two groups. The presence of at least moderate PVL was also not significantly different between the two groups at 1 month and 1 year (4% vs. 6%, *p* = 0.74). Average LVEF at 1 month was 30% ± 13 for the VLEF group vs. 40.2% ± 14 for the LEF group. The average change in EF at 1 month was 10% for the VLEF group and 9% for the LEF group (*p* = 0.89). Overall, 13/52 (25%) of patients in the VLEF group were re-hospitalized within 30 days of discharge vs. 51/257 (20%) of patients in the LEF group (*p* = 0.74). NYHA class III/IV symptoms and KCCQ scores were also similar among groups at both 1 month and 1 year (Table 3).

### 3.4. Subgroup Analysis within VLEF Group (Table 4)

A total of 29/55 (53%) of patients in the VLEF group received an SEV, while 26/55 (47%) received a BEV. Pre-valve BAV was performed in 9/29 (31%) of SEV recipients versus 10/26 (38%) of BEV recipients (*p* = 0.58). Post-valve BAV was required in 8/29 (28%) of SEV recipients vs. 3/26 (12%) of BEV recipients (*p* = 0.18). Moreover, 3/29 (10%) SEV patients required IABP use during the procedure for hemodynamic support, while none of the patients who received a BEV needed one (*p* = 0.24). Pacemaker rates were similar among groups (5/29 (17%) of SEV patients and 5/26 (19%) of BEV patients, *p* = 0.99). ECMO support was needed in only one patient, who received a SEV. Furthermore, 6/29 (21%) SEV recipients vs. 3/26 (12%) of BEV recipients experienced hypotension during TAVR (*p* = 0.47), and 8/29 (28%) of SEV recipients vs. 5/26 (19%) of BEV recipients required ≥1 vasopressor for hypotension lasting ≥30 min during procedure (*p* = 0.54) (Table 4).

Stroke rates were similar among groups: 1/29 (3% in the BEV vs. 1/26 (4%) in the SEV group, *p* = 0.99). Presence of moderate PVL, although numerically higher in the BEV group, was also not statistically different among groups (2/29 (7%) vs. 1/29 (24%), *p* = 0.99). LOS for SEV recipients was 9 ± 3 days vs. 5.5 ± 2 for BEV recipients (*p* = 0.03). Further, 29/29 SEV recipients and 25/26 BEV recipients were alive at the time of discharge. At one month, 26/29 (93%) of SEV recipients were alive. Two more patients who received BEV died within 1 month post procedure. Therefore, 23/26 (96%) of BEV recipients were alive at 1 month post procedure. The difference between both groups was not significant, as average LVEF at 1 month for SEV recipients was 29 ± 9% (n = 22) vs. 31 ± 17% (n = 21) for BEV recipients (*p* = 0.62). The change in EF for SEV recipients at 1 month was 11% (5, 20) for SEV recipients vs. 7.5% (0, 22) for BEV recipients (*p* = 0.53). Moreover, 6/25 (46%) of SEV recipients vs. 7/27 (54%) of BEV recipients required rehospitalization within 30 days of discharge (*p* = 0.99).

## 4. Discussion

Patients with severe AS and systolic dysfunction have a worse prognosis than patients with preserved left ventricular function. TAVR has been introduced as an attractive alternative to surgical aortic valve replacement for such patients [3,4]. Our multi-center patient cohort shows that TAVR is a valid option in this high-risk population with severe calcific AS and systolic CHF with severely reduced LVEF (≤20%). Our study found that the primary composite endpoint of in-hospital mortality, moderate or greater paravalvular (PVL), stroke, conversion to open surgery, aortic valve re-intervention, and/or need for PPM was not statistically different in patients with low vs. very low EF. Among patients with VLEF, we found that 30-day and 1-year survival was still above 90%. We also analyzed selection of THV among these patients with severely reduced EF and found that clinical outcomes did not differ despite the choice of SE vs. BE THV use.

### 4.1. TAVR in Low EF vs. Very Low EF

The PARTNER trial showed that TAVR is feasible alternative in symptomatic severe AS patients with LV dysfunction who were at high risk for surgical valve replacement [7]. Since then, TAVR has been shown to a safe and effective treatment option in patients across the entire spectrum of surgical risk despite left ventricular (LV) systolic dysfunction [8]. Prior studies have also showed that in patients with LVEF < 40%, almost half of them experience an improvement in EF by at least 10% within 30 days after TAVR [9,10]. Recently, Dhaval Kolte et al. published data on the 5-year all-cause death and cardiac death for a cohort of high- or intermediate-risk patients with severe AS and EF < 50% from the PARTNER trial, showing lower mortality in patients who experienced an improvement in LVEF after TAVR [11].

In accordance with published literature, TAVR in our population was associated with improvement of LVEF and NYHA functional class [12]. This was true for patients in both groups, with over 90% of patients demonstrating a change in EF at 1 month, with the average percentage of improvement in EF being approximately 10%. NYHA functional class also improved for both sets of patients. This improvement was most dramatic among patients in the VLEF group: 96% of patients had Class III/IV symptoms at baseline vs. only 22% at 1 year post TAVR. A good corollary of symptoms is the KCCQ questionnaire score, and it improved significantly from baseline for both groups post TAVR. There was no significant difference in KCCQ scores at 1 month or 1 year between the LEF and the VLEF groups.

The lack of significant difference between the two groups in the primary and secondary outcomes was surprising since we noted a more complex periprocedural course in patients with VLEF. Patients in the VLEF were more prone to use of inotropic agents and IABP during TAVR. Moreover, the rate of blood transfusions was higher in the VLEF group. Given the more complex periprocedural course of patients in the VLEF group, their length of stay was significantly higher when compared to patients in the LEF group. However, stroke rates and mortality at time of discharge were not significantly different between the two groups. While it seems that the periprocedural period was more complicated in patients with very low EF, they had successful short- and intermediate-term outcomes.

A concern with TAVR in patients with systolic dysfunction remains the possible need for ventricular pacing. New PPM placement can have a negative effect on LVEF that impacts survival [13]. Our data did not show a difference in rates of PPM placement between VLEF and LEF. This was also true when comparing outcomes of patients who received an SEV vs. those who received a BEV in either group. Interestingly, rates of PPM were higher for BEV than in previous randomized control studies. This may be related to bias because patients at higher risks for conduction deficits likely received BEV. Furthermore, patients with pre-existing myopathy may have higher conduction deficits post TAVR. The rates of moderate to severe PVL were numerically higher in patients with LEF, but the difference was not statistically significant. Half of the patients in either group with moderate/severe PVL did not survive to 1 year, and there was poor echocardiographic follow-up (~50% of patients). Therefore, the lack of significance at 1 year in PVL rates may be due to attrition bias or selection bias.

Length of stay was longer in our study patients than contemporary trials, whereby the median LOS for TAVR was ~2 days [8]. Our longer LOS may be attributed to our sicker patient population (low LVEF and with more comorbidities). There was also a significant preoperative LOS, suggesting that many of these patients were either inpatient at the time of TAVR or required preoperative rehabilitation prior to procedure. Nonetheless, we found that there was a reduction in LOS by ~4 days in the BEV group. One potential explanation for this is the numerically higher PPM insertion in SEV (which typically adds an extra day at least in the hospital). Another explanation may be that they had a worse degree at baseline (NYHA Class III/IV)). This suggests that the periprocedural course is similar in this patient population despite the valve choice.

### 4.2. Choice of THV among Patients with Very Low EF

The hemodynamic effects of THV choice may be more significant in patients with systolic heart failure. The percentage of patients with NYHA III/IV at one month or one year post TAVR was not statistically different between the LEF and VLEF. However, as expected, there were more patients from the VLEF group with NYHA III/IV symptoms at one year. This is likely due to the progression of cardiac disfunction and comorbid conditions for these patients. While advanced NYHA class was more common in the SEV group (NYHA IV) at baseline, NYHA III/IV symptoms were numerically more common in the BEV group on follow-up at 1 month and 1 year. Due to the small number of patients, we cannot make any conclusions regarding why patients in the BEV group had more NYHA III/IV symptoms at follow-up. A longer study with more patients is required to determine if the numerical difference of patients with more severe symptoms would be statistically significant.

The periprocedural outcomes were not significantly different between BEV and SEV in this population with very low EF except for length of stay. Length of stay was longer after SEV TAVR vs. BEV TAVR. This is likely because of the longer length of stay for the patients who had IABP placement and the one patient who had ECMO placement. These four patients had significant baseline comorbidities and an extended hospital course prior to TAVR. Due to the hemodynamic profile of these patients and their anatomy, a multidisciplinary team decision was made to perform TAVR rather than just BAV despite their significant morbidity.

## 5. Limitations

Our study was limited by its non-randomized nature. We included both older- and newer-generation THV in our analysis. The choice of THV was made by each physician, and the reason for THV choice is not included in our study. Patients with NYHA III/IV more commonly received a SEV. This may reflect a potential concern for the use of BEV due to the need for rapid pacing and associated hypotension. However, our subgroup analysis was not powered to evaluate the differences in outcomes between recipients of BEV vs. recipients of SEV. Lastly, the follow-up period of 1 year may not be enough to see a difference in clinical outcomes between the two groups since we know from analysis of the PARTNER 2 trial that baseline EF is an independent predictor of 2-year cardiovascular mortality.

## 6. Conclusions

In patients with ejection fraction ≤20%, TAVR can be a safe procedure associated with good short-term and intermediate-term results. Although the perioperative period is more challenging for these patients compared to those with higher EF, the clinical outcomes are not significantly different at discharge or within one year. The choice of THV must be weighed on a case-by-case basis. but our initial assessment suggests that BEVs and SEVs are associated with similar outcomes in this population. Larger studies are needed to confirm these results.

## Figures and Tables

**Table 1:** Baseline Characteristics.

Characteristic	EF ≤ 20%	20% < EF ≤ 40%	*p*-Value
Age	79.8 ± 9.6	81.1 ± 8.5	0.33
Female (%)	22/55 (40)	85/280 (30%)	0.21
Prior CIED/PPM (%)	22/55 (40)	80/280 (29%)	0.11
Prior PCI/CABG (%)	23/55 (42)	161/280 (57%)	0.04
Other cardiac surgery (%)	9/55 (16)	56/280 (20%)	0.71
DM (%)	21/55 (38)	112/280 (40%)	0.88
PVD (%)	18/55 (33)	71/280 (25%)	0.32
Baseline Cr	1.3 ± 0.7	1.6 ± 1.4	0.1
Baseline Mg	33 ± 13	35 ± 14	0.29
Baseline KCCQ	39 ± 24	47 ± 24	0.02
STS	11.0 ± 7.9	9.3 ± 7.3	0.12
Smoking (%)	3/55 (5)	12/280 (4%)	0.72
Baseline hemoglobin	11.8 ± 1.7	11.8 ± 1.8	0.79
Prior stroke (%)	1/55 (2)	28/280 (10%)	0.06
Corevalve (%)	29/55 (53)	117/280 (42%)	0.14
Baseline NYHA III/IV (%)	53/55 (96)	236/280 (84%)	0.02
Baseline NYHA I/II) (%)	2/55 (3.6%)	44/280 (16%)	0.02
Baseline peak gradient (mmHg)	54 ± 20	59 ± 22	0.18
Baseline AVA (cm^2^)	0.63 ± 0.17	0.69 ± 0.25	0.06
Mean AV gradient (mmHg)	31.621 ± 12.588	35.214 ± 14.225	0.67

**Table 2:** Periprocedural outcomes.

	EF ≤ 20	20 < EF ≤ 40	*p*-Value
Primary composite endpoint	14/55 (24%)	55/280 (20%)	0.47
Discharge deceased (%)	1/55 (2%)	5/280 (2%)	0.99
≥Moderate PVL (%)	1/55 (2%)	14/280 (5%)	0.48
Stroke (%)	1/55 (2%)	4/280 (1%)	0.59
Conversion to surgery	1/55 (2%)	0/280 (0%)	0.16
Aortic re-intervention	1/55 (2%)	1/280 (<1%)	0.30
Need for PPM (%)	10/55 (18%)	48/280 (17%)	0.85
IABP use (%)	5/55 (9%)	2/280 (1%)	<0.01
ECMO	1/55 (2%)	0/280 (0%)	0.16
Transfusion (%)	21/55 (38%)	59/280 (21%)	<0.01
Use of 2+ pressors intraop.	22/55 (40%)	60/280 (21%)	<0.01
Total LOS	6 ± 3 Days	3 ± 2 Days	<0.01

**Table 3:** One Month and One Year Outcomes.

	EF ≤ 20	20 < EF ≤ 40	*p*-Value
Mortality at 1 month	5/55 (9%)	13/280 (5%)	0.19
Mean AVG at 1 month	11 ± 10, *n* = 45	9 ± 5, *n* = 224	0.07
≥Moderate PVL at 1 month	2/49 (4%)	14/220 (6%)	0.74
1M LVEF (%)	30% ± 13, *n* =49	40.2% ± 14, *n* = 220	<0.01
1M average change in EF (%)	10%	9%	0.89
KCCQ score at 1 month	67 ± 24, *n* = 34	69 ± 26, *n* = 168	0.74
NYHA III/IV at 1 month	3/52 (6%)	34/257 (13%)	0.54
Rehospitalization at 1M	13/52 (25%)	51/257 (20%)	0.74
Mortality at 1 year	7/55 (13%)	30/280 (11%)	0.64
Mean AVG at 1 year	12 ± 9, *n* = 22	11 ± 8, *n* = 122	0.48
≥Moderate PVL at 1 year	3/24 (12%)	3/116 (5%)	0.18
KCCQ score at 1 year	77.54 ± 20.43 (*n* = 18)	74.97 ± 23.58 (*n*= 127)	0.56
NYHA III/IV classification at 1 year	4/18 (22%)	9/127 (7%)	0.06

**Table 4:** Subgroup Analysis within VLEF Group Based on Valve Type.

Characteristic	Self-Expandable Valve (SEV)	Balloon-Expandable Valve (BEV)	*p*-Value
Type of valve placed during TAVR	29/55 (53%)	26/55 (47%)	
	Corevalve: 4	Sapien XT: 3	
	Evolut R: 18	Sapien 3: 23	
	Evolut Pro: 6		
	Evolut Pro+: 1		
Intra-aortic balloon pump (IABP)	3/29 (10%)	0/26 (0%)	0.24
Permanent pacemaker (PPM) after TAVR	5/29 (17%)	5/26 (19%)	0.99
Extracorporeal membrane oxygenation (ECMO)	1/29 (3%)	0/26 (0%)	0.99
Pre-valve balloon aortic valvuloplasty (BAV)	9/29 (31%)	10/26 (38%)	0.58
Post-valve balloon aortic valvuloplasty (BAV)	8/29 (28%)	3/26 (12%)	0.18
Hypotension during TAVR	6/29 (21%)	3/26 (12%)	0.47
Vasopressor support during TAVR	8/29 (28%)	5/26 (19%)	0.54
Stroke	1/29 (3%)	1/26 (4%)	0.99
≥Moderate paravalvular leak (PVL)	2/29 (7%)	1/29 (4%)	0.99
Length of stay (LOS), days	9 ± 3	5.5 ± 2	0.03
Alive at time of discharge	29/29 (100%)	25/26 (96%)	0.47
Alive at 1 month post discharge	26/29 (93%)	23/26 (88%)	0.99
Average LVEF at 1 month post discharge (%)	29 ± 9 (*n* = 22)	31 ± 17 (*n* = 21)	0.62
Change in LVEF at 1 month (%)	11 (5, 20)	7.5 (0, 22)	0.53
Rehospitalization within 30 days of discharge	6/25 (46%)	7/27 (54%)	0.99

## Data Availability

Data sharing not applicable.
